# Association of apolipoprotein E (*APOE*) polymorphisms with warfarin maintenance dose in a northern Han Chinese population

**DOI:** 10.1186/s12944-016-0205-8

**Published:** 2016-02-24

**Authors:** Rui Liu, Kui Zhang, Zhi-zhong Gong, Xin-miao Shi, Qian Zhang, Xiao-dong Pan, Ran Dong

**Affiliations:** Cardiac Surgery, Beijing Institute of Heart, Lung and Blood Vessel Disease, Beijing Anzhen Hospital, Capital Medical University, No. 2 Anzhen Street, Chaoyang District Beijing, 100029 China; Department of Epidemiology, Beijing Institute of Heart, Lung and Blood Vessel Disease, Beijing Anzhen Hospital, Capital Medical University, No. 2 Anzhen Street, Chaoyang District Beijing, 100029 China; Division of Cardiology, Department of pediatrics, Shandong Provincial Hospital Affiliated to Shandong University, Jingwuweiqi Street, Huaiyin District Jinan, 250000 China; Experimental Center, Beijing Institute of Heart, Lung and Blood Vessel Disease, Beijing Anzhen Hospital, Capital Medical University, No. 2 Anzhen Street, Chaoyang District Beijing, 100029 China

**Keywords:** Apolipoprotein E, Gene polymorphism, Thromboembolism, Warfarin therapy, Northern Han Chinese patients, Heart valve prosthesis

## Abstract

**Background:**

Apolipoprotein E (apoE) induces the uptake of vitamin K-rich lipoproteins by the liver, which likely affects inter-individual variation of warfarin dosing requirements. Associations between *APOE* polymorphisms and warfarin dosing were previously reported inconsistently among different ethnic groups, so the present study investigated this association in northern Han Chinese patients with mechanical heart valve prosthesis.

**Methods:**

A total of 186 patients who underwent mechanical heart valve replacement and attained a stable warfarin dose were included. *APOE* single nucleotide polymorphisms (SNPs) rs7412 and rs429358 were genotyped using Illumina SNP GoldenGate Assay. Genotyping results were confirmed by direct sequencing. PHASE v2.1 software was used to construct rs7412 and rs429358 haplotypes. The effects of different *APOE* genotypes on warfarin dose were analyzed statistically.

**Results:**

The mean warfarin maintenance dose was 3.10 ± 0.96 mg/day, and the mean international normalized ratio (INR) was 2.09 ± 0.24. *APOE* E2, E3, and E4 allele frequencies were 11.6 %, 82.5 %, and 5.9 %, respectively. No E2/E2 or E4/E4 genotypes were detected in this population. E2/E3, E3/E3, E2/E4, and E3/E4 genotype frequencies were 21.0 %, 67.2 %, 2.2 %, and 9.7 %, respectively. Significant differences in warfarin dose requirements were observed among patients with E2/E3, E3/E3, and E3/E4 genotypes (*p* < 0.05). In post hoc comparison, daily warfarin maintenance doses were significantly higher in E2/E3 heterozygotes compared with E3/E3 homozygotes (*p* < 0.05), but no differences in dose requirements were found between E3/E4 and E3/E3, or E2/E3 and E3/E4 (*p* > 0.05). Patients were divided into low-intensity anticoagulant treatment group (1.6 ≤ INR <2.0) and relatively high-intensity anticoagulant treatment group (2.0 ≤ INR ≤2.5), and significantly higher warfarin dose requirements were observed in E2/E3 heterozygotes compared with E3/E3 homozygotes in both subgroups (*p* < 0.05). Multivariable analysis adjusting for other confounders showed that E2/E3 genotype was associated with a significantly higher warfarin dose compared with E3/E3 genotype (*p* < 0.05).

**Conclusions:**

*APOE* allele and genotype frequencies in the northern Han Chinese population appear to differ from other racial groups or populations living in other regions of China. The *APOE* E2 variant was associated with a significantly higher warfarin maintenance dose. Thus, *APOE* polymorphisms could be one of the predictors influencing warfarin doses in this population.

## Background

Thromboembolisms, especially cerebral embolisms, are a common complication of many cardiovascular and cerebrovascular diseases such as atrial fibrillation, heart valve prosthesis, deep venous thrombosis, and stroke. Warfarin is the most highly prescribed oral anticoagulant for the treatment and prevention of thromboembolism in clinical practice. However, because of its narrow therapeutic index and the wide variability in drug response among individuals, its use is reported to be the most common adverse drug event, accounting for approximately one third of emergency hospitalizations for the elderly [1, 2]. Thus, prescribing the correct dosage of warfarin is directly related to the prognosis of many cardiovascular and cerebrovascular diseases.

Several studies have shown that genetic polymorphisms are the main factor responsible for inter-individual variability in warfarin response. Because of this, the US Food and Drug Administration revised the drug instructions in 2007 and 2010 to incorporate genetic information about *VKORC1* and *CYP2C9* to guide the clinical medication of warfarin [3]. The International Warfarin Pharmacogenetics Consortium also explicated a pharmacogenetic model incorporating *VKORC1*, *CYP2C9*, and nongenetic factors such as age, height, weight, and the combination use of amiodarone to predict warfarin dose in clinical practice [4]. However, this pharmacogenetic algorithm and others can only explain approximately 50 % of warfarin dose variation [5–7], indicating that additional genetic polymorphisms may be involved in affecting warfarin dosing requirements.

Apolipoprotein E (APOE) is an essential component of lipoproteins that is involved in lipid transportation and induces the uptake of lipoproteins rich in vitamin K1 by the liver [8]. Because warfarin exerts its anticoagulant effects by preventing oxidized vitamin K from regenerating reduced vitamin K in the liver, warfarin dose requirements are likely to be influenced by the availability of hepatic vitamin K. Human *Apoe*, situated on chromosome 19, exists in three common allelic forms: E2, E3, and E4, giving six possible genotypes (E2/E2, E2/E3, E2/E4, E3/E3, E3/E4, and E4/E4) [9–11]. In previous studies, associations between *APOE* polymorphisms and warfarin dosing were reported inconsistently among different ethnic groups. Given that the northern Han Chinese population differs in terms of lifestyle, culture, and dietary habits from those in other regions of China, it is meaningful to investigate the potential association between *APOE* polymorphisms and warfarin dosing in northern Han Chinese patients with mechanical heart valve prosthesis.

## Results

### Characteristics of the study population

Demographic and clinical characteristics of patients (*n* = 186) are shown in Table 1. Smoking, drinking, as well as BSA, weight, height, blood urea nitrogen (BUN), and serum creatinine (SCR) showed significant differences between males and females (*p* < 0.05), but no difference was found among other variables (*p* > 0.05).

**Table 1:** Comparison of demographic and clinical characteristics in terms of gender

Variable	Males	Females	*P*-value
Age (years)	54.95±11.15	56.42±9.08	0.328
Smoking (n, %)	8(8.6)	0(0)	0.004
Drinking (n, %)	8(8.6)	0(0)	0.004
BSA (m^2^)	1.78±0.16	1.61±0.13	<0.001
BMI (kg/ m^2^)	24.20±3.18	24.01±3.27	0.686
Weight (kg)	70.12±10.81	61.43±9.14	<0.001
Height (cm)	170.08±5.61	159.89±5.15	<0.001
Hypertension (n, %)	21(22.6)	12(12.9)	0.084
Hyperlipidemia (n, %)	10(10.8)	8(8.6)	0.620
Diabetes (n, %)	7(7.5)	11(11.8)	0.321
CAD (n, %)	9(9.7)	8(8.6)	0.799
Stroke (n, %)	4(4.3)	4(4.3)	1.000
SBP (mm Hg)^*^	125 (120–130)	125 (116.5–130)	0.990
DBP (mm Hg)^*^	74 (64–80)	74 (64–80)	0.737
Triglycerides (mmol/L)^*^	1.31 (0.86–2.05)	1.30 (0.99–1.87)	0.540
Total cholesterol (mmol/L)	4.72±1.02	4.81±1.13	0.566
LDL-C (mmol/L)	2.92±0.86	2.96±0.91	0.782
HDL-C (mmol/L)	1.11±0.31	1.15±0.32	0.342
FBG (mmol/L)^*^	5.28 (4.91–5.74)	5.40 (4.97–5.91)	0.398
CRP (mg/L)^*^	1.01 (0.47–3.35)	1.11 (0.59–3.47)	0.638
BUN (mmol/L)^*^	6.70 (5.80–7.85)	5.50 (4.70–6.70)	<0.001
SCR (umol/L)	83.25±13.60	69.71±19.93	<0.001
eGFR (ml/min/1.73m^2^)	94.67±16.52	89.78±19.39	0.066
Amiodarone use (n, %)	2(2.2)	4(4.3)	0.678
Diltiazem use (n, %)	1(1.1)	4(4.3)	0.365
Statins use (n, %)	8(8.6)	5(5.4)	0.388
Dioxin use (n, %)	17(18.3)	16(17.2)	0.848
Insulin use (n, %)	4(4.3)	5(5.4)	0.733

*Continuous variables with skewed distribution were reported as medians (Q1 and Q3); BSA: body surface area; BMI: body mass index; CAD: coronary artery disease; SBP: systolic blood pressure; DBP: diastolic blood pressure; HDL-C: high-density lipoprotein cholesterol; LDL-C: low-density lipoprotein cholesterol; FBG: fasting blood glucose; CRP: C-reactive protein; BUN: blood urea nitrogen; SCR: serum creatinine; eGFR: glomerular filtration rate

### Genotyping

**Fig. 1:**
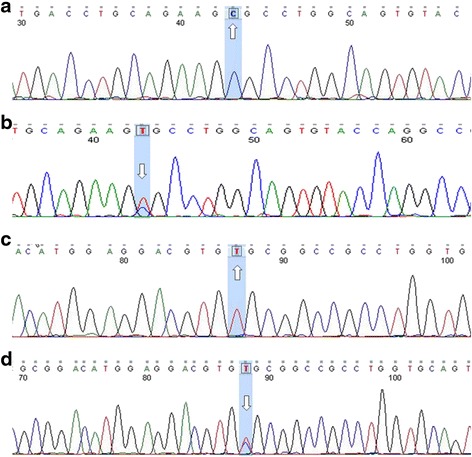
Sequence of *APOE* rs7412 (CC, CT) and rs429358 (TT, TC) genotypes (5’-3’), the arrow showed the mutation. (**a**: rs7412 CC, **b**: rs7412 CT, **c**: rs429358 TT, **d**: rs429358 TC)

As shown in Fig. 1, genotyping results for rs7412 and rs429358 were confirmed by direct sequencing. PHASE v2.1 software was used to construct haplotypes of *APOE* single nucleotide polymorphisms (SNPs) rs7412 and rs429358. Haplotype TT was coded as E2, TC as E3, and CC as E4 alleles. E2, E3, and E4 allele frequencies were 11.6 %, 82.5 %, and 5.9 %, respectively. As shown in Table 2, we observed a difference between our data from the northern Han Chinese population and those from previous studies of other ethnic groups. The observed genotype frequency showed no deviation from Hardy–Weinberg equilibrium (HWE; χ^2^ = 4.342, *p* = 0.227).

**Table 2:** *APOE* allele and genotype frequencies obtained in the study population and in other ethnic groups

Gene	Allele	Frequency (N, %)	Genotype	Frequency (N, %)	Reference genotype frequency in other ethnic groups (N, %)				
					Southwest Chinese [16]	Caucasians [17]	African -American [17]	Egyptians [24]	Brazilians [18]
APOE	E2	43(11.6)	E2/E2	0(0)	3(1.20)	0(0)	3(2.7)	3(1.54)	1(0.8)
	E3	307(82.5)	E2/E3	39(21)	39(15.66)	16(13.22)	15(13.51)	20(10.26)	17(14.7)
	E4	22(5.9)	E2/E4	4(2.2)	2(0.80)	3(2.48)	7(6.31)	3(1.54)	2(1.7)
			E3/E3	125(67.2)	180(72.29)	73(60.33)	51(45.95)	146(74.87)	66(56.9)
			E3/E4	18(9.7)	23(9.24)	28(23.14)	30(27.03)	23(11.79)	26(22.5)
			E4/E4	0(0)	2(0.80)	1(0.83)	5(4.50)	0(0)	4(3.4)

### Effect of *APOE* polymorphisms on warfarin maintenance dose

The stable warfarin dose and INR values of patients with different *APOE* genotypes are shown in Table 3. Mean stable warfarin dose requirements were 3.10 ± 0.96 mg/day (range, 0.75–7.5 mg/day), with a coefficient of variance of 30.97 %. Significant differences in warfarin dose requirements were observed among carriers of E2/E3, E3/E3, and E3/E4 genotypes (*p* < 0.05). In the post hoc pairwise comparison, warfarin dose requirements were significantly higher in patients with the E2/E3 genotype compared with those with the E3/E3 genotype (*p* < 0.05), but no differences were found between carriers of E3/E4 and E3/E3, or E2/E3 and E3/E4 genotypes (*p* > 0.05). In addition, the mean INR values were 2.09 ± 0.24, but there was no significant difference in INR values among the three *APOE* genotypes (*p* > 0.05).

**Table 3:** Daily warfarin maintenance dose and INR values of different *APOE* genotypes

APOE genotype	Patient Number	Mean ± SD INR value^b^	Mean ± SD warfarin dose (mg/d)	*P* value
E2/E3 (E2 carrier^a^)	39	2.06±0.25	3.47±1.04	0.024
E3/E3	125	2.10±0.24	2.99±0.93	
E3/E4 (E4 carrier^a^)	18	2.11±0.21	2.97±0.82	
E2/E3 vs E3/E3				0.007
E2/E3 vs E3/E4				0.080
E3/E3 vs E3/E4				0.993

^*a*^Patients (n=4) with the E2/E4 genotype were excluded from analysis

^*b*^ *p*=0.511 (compared among three genotypes)

**Fig. 2:**
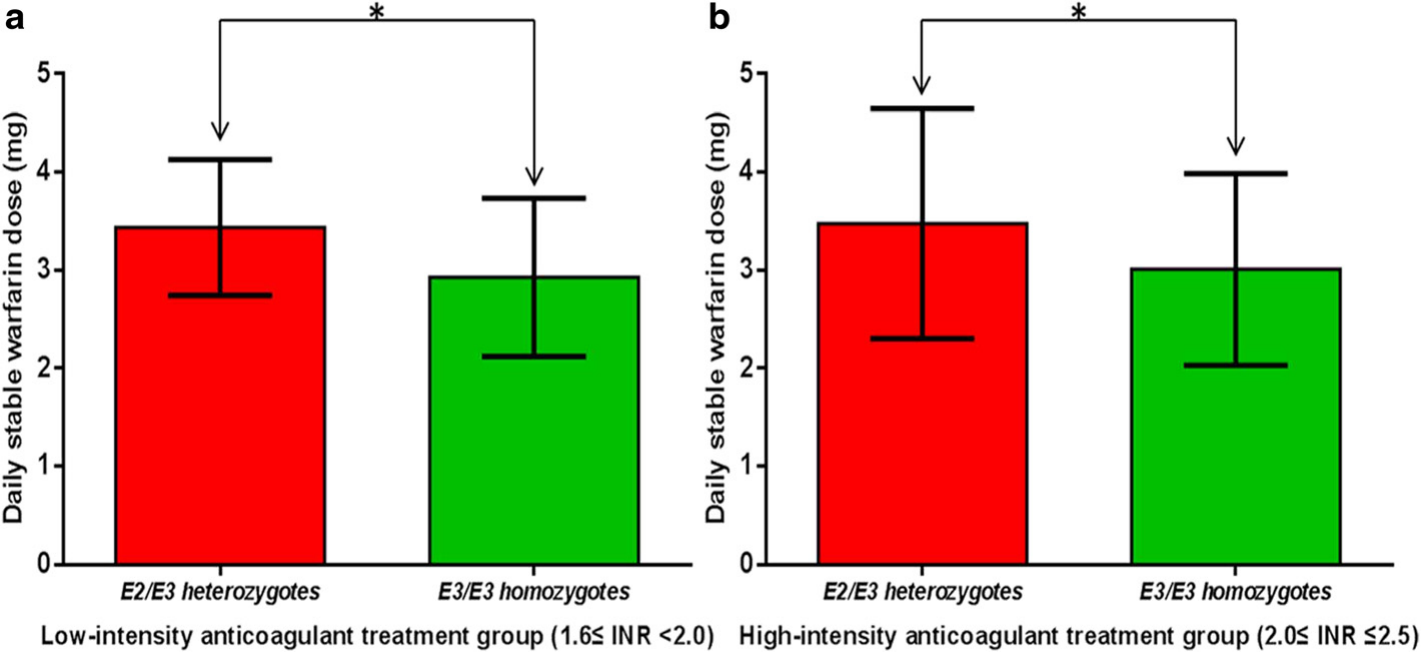
Comparison of daily stable warfarin dose requirements between E2/E3 heterozygotes and E3/E3 homozygotes in two subgroups based on patient INR values. (**a**) low-intensity anticoagulant treatment group: 1.6 ≤ INR <2.0; (**b**) relatively high-intensity anticoagulant treatment group: 2.0 ≤ INR ≤2.5, * *p* < 0.05, analyzed by independent *t*-tests

Considering that patients with different heart valve prostheses require different treatment goals, we divided patients into low-intensity anticoagulant treatment group (1.6 ≤ INR <2.0) and relatively high-intensity anticoagulant treatment group (2.0 ≤ INR ≤ 2.5) based on measured INR values. As shown in Fig. 2, stable warfarin dose requirements were significantly higher in E2/E3 heterozygotes compared with E3/E3 homozygotes in both subgroups (both *p* < 0.05).

After adjusting for other variables (sex, age, BSA, BMI, eGFR categories, smoking and drinking status, coronary artery disease, hyperlipidemia, hypertension, diabetes, stroke history, measured INR and number of concomitant medications that increase INR values) in a multivariable analysis, E2/E3 genotype was associated with a statistically higher warfarin dose compared with E3/E3 genotype (*p* < 0.05). Besides, BSA was associated with a significantly higher warfarin dose (*p* < 0.05) while increased age and number of concomitant medications that increased INR values were associated with a significantly lower warfarin dose (*p* < 0.05) (Table 4).

**Table 4:** Multivariable models for *APOE* polymorphisms using square root transformed warfarin dose

Variables	Beta Coefficients	*P*-value
Intercept	1.515	
Age (per year increase)	-0.006	0.002
Body surface area	0.325	0.005
*E2/E3* genotype^*^	0.113	0.020
*E3/E4 genotype* ^*^	-0.018	0.782
Number of concomitant medications that increase INR values^†^	-0.130	0.035

^*^We set the E3/E3 genotype as a reference, and regarded E2/E3 and E3/E4 genotype as dummy variables in the multivariable analysis. ^†^Concomitant medications included amiodarone, diltiazem, fluvastatin, simvastatin and lovastatin

## Discussion

In the present study, we demonstrated *APOE* E2, E3, and E4 allele frequencies in the northern Han Chinese population of 11.6 %, 82.5 %, and 5.9 %, respectively, which differs from data of other ethnic groups. The allele frequency of E2 (11.6 %) in this study was higher than the results reported by Huang et al [12] derived from the southwest Chinese population. The E4 allele frequency (5.9 %) was similar to that reported by Huang et al, but lower than that of other ethnic groups, which ranged from 13.64 % to 21.17 % [13, 14]. Significant differences in warfarin dose requirements were observed among carriers of E2/E3, E3/E3, and E3/E4 genotypes. In post hoc comparison, E2/E3 heterozygotes required a 16.1 % higher daily warfarin dose than E3/E3 homozygotes. No differences in dose requirements were found between carriers of E3/E4 and E3/E3, or E3/E4 and E2/E3 genotypes. Based on differences in measured INR values, we divided patients into low-intensity warfarin treatment group (1.6 ≤ INR <2.0) and relative high-intensity warfarin treatment group (2.0 ≤ INR ≤2.5). Significantly higher warfarin dose requirements were observed in E2/E3 heterozygotes compared with E3/E3 homozygotes in both subgroups (*p* < 0.05). In a multivariable analysis adjusting for other demographic and clinical variables such as age, sex, BSA, eGFR categories and number of concomitant medications that potentiate warfarin, E2/E3 genotype was associated with a significantly higher warfarin does compared with E3/E3 genotype (*p* < 0.05). To our knowledge, this is the first report linking *APOE* polymorphisms with the warfarin dosage in a Chinese population.

To date, the effect of *APOE* polymorphisms on the anticoagulant response and dose requirements of warfarin have been reported inconsistently. APOE induces the uptake of vitamin K-rich lipoproteins by the liver. Changes in vitamin K content in the body can significantly affect warfarin anticoagulation activity and dose requirements, but the mechanism by which *APOE* polymorphisms acts on liver vitamin K availability has been poorly elucidated. Because different *APOE* isoforms exhibit different affinities to remnant and LDL receptors, the hepatic clearance of vitamin K-rich chylomicron remnants varies between *APOE* isoforms and is in the order of E2 < E3 < E4. Thus, it has therefore been hypothesized that the E2 variant mediates a relatively low uptake of vitamin K by the liver, inhibiting the hepatic availability of vitamin K for the carboxylation of clotting factors. Thus, apoE2 carriers theoretically require less warfarin to achieve adequate anticoagulation effects. However, in a contrasting hypothesis, E3 and E4 variants exhibit an accelerated clearance of vitamin K-rich chylomicrons from the blood compared with the E2 variant. This implies an accelerated uptake of vitamin K by the liver. However, most accelerated vitamin K in the liver bypasses the intracellular vitamin-K dependent synthesis of active coagulation factors, but facilitates the more extensive metabolism and excretion and is directly eliminated. As a consequence, E3 and E4 variants lead to a decreased hepatic availability of vitamin K, so apoE3 or apoE4 carriers require less warfarin than apoE2 carriers. Our results support the latter hypothesis.

Our results are similar to those of Huang et al. [12] from a southwest Han Chinese population, which indicated that patients carrying the E2 allele required a higher warfarin maintenance dose. Sconce et al. [8] also reported similar results from a Caucasian population. However, both of these studies failed to show a statistically significant difference, unlike our present findings. Besides, Sconce et al. showed that patients carrying at least one E4 allele required a lower warfarin maintenance dose than those carrying the E3/E3 genotype (*p* < 0.05). Results from de Oliveira et al. [14] in Brazilian patients also demonstrated that patients carrying at least one E4 allele required a lower warfarin dosage. By contrast, Kohnke et al. studied a Swedish population [15] and suggested that E4 carriers (E2/E4, E3/E4, and E4/E4 genotypes) have enhanced liver uptake of vitamin K and require a higher warfarin dosage to compensate for this. Our study found that patients carrying the E4 allele showed no significant difference in dose requirements from E3/E3 or E2/E3 genotype carriers. This is likely due to the fact that the E4 allele frequency is much lower in the Chinese population compared with other ethnic groups. Because only 18 patients carrying the E3/E4 genotype were detected in the present study, it was difficult to establish whether the *APOE* E4 variant influenced warfarin maintenance dose in the northern Han Chinese population.

The present study has a number of limitations. First, we did not measure plasma vitamin K concentrations, yet different concentrations may reflect the delivery efficacy of vitamin K-rich remnants to the liver in different *APOE* isoforms. Second, our study was restricted by its relatively small sample size; in particular, only 18 patients had the E3/E4 genotype and no E2 or E4 homozygotes were detected. Finally, because we recruited patients with heart valve prosthesis from the northern China, our results may not be able to be directly generalized to other populations.

## Conclusions

*APOE* allele and genotype frequencies in the northern Han Chinese population differ from those of other racial groups and populations living in other regions of China. *APOE* E2/E3 genotype was associated with a significantly higher warfarin maintenance dose compared with E3/E3 genotype. Thus, *APOE* polymorphisms appear to be one of the predictors influencing warfarin dose in the northern Han Chinese population. The effect of *APOE* polymorphisms on warfarin dosage was relatively small, thus consideration of the *APOE* genotype in isolation is unlikely to be of clinical relevance. Whereas, if rapid and cost-effective genotyping technologies were available in the future, it would be feasible to test *APOE* genotypes, together with *VKORC1*, *CYP2C9* and other nongenetic factors, to help better predict warfarin dose requirements.

## Methods

### Subjects

Between April 2012 and May 2015, 226 ethnic Han Chinese inpatients undergoing mechanical heart valve replacement were recruited from the Cardiac Surgery Department of Anzhen Hospital, Capital Medical University (Beijing, China). Our inclusion criteria were that patients had to be Han Chinese and lived in northern China (Beijing, Shandong, Shanxi, Henan, Hebei, Heilongjiang, Jilin, Liaoning, and Inner Mongolia provinces); at least 18 years old; able to provide written consent; under warfarin anticoagulation for more than 3 months and had attained a stable maintenance dose. A stable warfarin maintenance dose was defined as administration of a constant dose for at least 1 month with international normalized ratio (INR) measurements within the target range for multiple time periods after discharge. The target ranges were 1.6–2.0 for aortic valve replacement, 1.8–2.5 for mitral valve replacement, and 1.8–2.5 for double valve replacement. This relatively low target INR was chosen because Asian patients are more sensitive to warfarin therapy so are more likely to suffer episodes of bleeding than Caucasians [16]. Thus, the majority of Chinese patients are suitable for low intensity anticoagulation with a target INR lower than 2–3. Compared with standard intensity anticoagulation, this lower intensity is equally effective at preventing thrombotic events, and more likely to decrease bleeding during treatment [17–20].

Our exclusion criteria were as follows: (1) hematological disease or hemorrhagic tendencies; (2) blood platelet count <120 × 10^9^/L; (3) heart failure (New York Heart Association class 3 or class 4); (4) liver dysfunction (defined as the presence of chronic hepatic disease or biochemical evidence of significant hepatic impairments); (5) renal dysfunction (defined as chronic dialysis or serum creatinine >200 mM or recipient of kidney transplantation); (6) thyroid disease; (7) malignant tumors; (8) peptic ulcers; (9) infections; (10) autoimmune disease; and (11) pregnancy. This study was carried out in accordance with the Declaration of Helsinki Principles and approved by the Ethics Committee of Beijing Anzhen Hospital, Capital Medical University. All subjects provided written informed consent to participate in the study.

The patients were followed up after surgery until August 2015, and the follow-up period ranged from 4 to 35 months. During follow-up, 17 patients who did not meet the criteria were excluded from study. 17 patients refused to participate after enrollment. One patient died of unexplained heart failure. Three patients lost to follow up. Another two patients who were unsuccessfully genotyped were also excluded from study. Thus, a total of 186 patients with complete genetic and clinical information were included in subsequent analysis.

### Clinical data

We collected patient clinical data by means of regular telephone calls, face-to-face interviews, and a review of medical records from our hospital information system. These data included age, gender, age at the time of operation, height, weight, indication for warfarin treatment, valve position, valve types, routine INR values, stable warfarin dose, concomitant disease, concurrent interacting medications, current smoking status, and alcohol consumption. Body surface area (BSA) was defined as: 0.0061 × height (cm) + 0.0128 × weight (kg) – 0.1529 [21], and body mass index (BMI) as: weight (kg)/ [height (m)^2^]. Glomerular filtration rate (eGFR) was estimated using the Chronic Kidney Disease Epidemiology Collaboration (CKD-EPI) equation [22] and grouped patients into the KDIGO (Kidney Disease: Improving Global Outcomes) eGFR categories [23]: ≥90, 60–89 and 45–59 ml/min/1.73 m^2^. Hyperlipidemia was diagnosed with an elevation of at least one of the following: >6.22 mmol/L for total cholesterol, >2.26 mmol/L for triglycerides, or >4.14 mmol/L for low-density lipoprotein cholesterol (LDL-C); hypertension was diagnosed after taking a mean of three independent measures of blood pressure >140/90 mmHg; and diabetes mellitus was diagnosed if the patient had a fasting glucose >7.0 mmol/L, or >11.1 mmol/L 2 h after an oral glucose challenge, or both. Smokers were self-reported as using cigarettes every day and having smoked in the past 30 days before follow-up; drinkers of alcohol were defined as those consuming a daily average of ≥15 g pure alcohol for females or ≥25 g for males during the past year.

### Biochemical parameters

Blood samples were drawn from each patient under morning fasting conditions. Triglycerides (TG), serum total cholesterol (TC), low density lipoprotein cholesterol (LDL-C), high density lipoprotein cholesterol (HDL-C), fasting blood glucose (FBG), blood urea nitrogen (BUN), serum creatinine (SCR) and other biochemical parameters were determined at the clinical laboratory of Beijing Anzhen Hospital, Capital Medical University.

### DNA extraction

Blood samples were taken from eligible patients for genotyping before they underwent surgery. A total of 5 ml venous blood from each patient was collected in an EDTA vacuum tube and stored at 4 °C before DNA extraction. Genomic DNA was extracted using the Blood DNA System DNA isolation kit (CWBIO, Beijing, China) according to the manufacturer’s instructions and stored at 4 °C for subsequent use. DNA quality and purity were assessed by agarose gel electrophoresis, and optical absorbance was measured at A260/A280.

### SNP beadchip assay

Genotyping was performed using the Illumina SNP Golden Gate Assay (Illumina, Inc., San Diego, CA) according to the manufacturer’s specifications. Briefly, 250 ng of genomic DNA was amplified at 37 °C for 20 h, then fragmented and precipitated. The dried pellet was resuspended and hybridized to the beadchip. Hybridized beadchips were incubated at 48 °C for 20 h, washed, and used for single-base extension. Beadchips were then stained, washed, coated, and dried. Finally, signal intensity data were generated by an Illumina BeadArray Reader. We randomly selected 20 % of all samples to be genotyped in duplicate, which resulted in 99.8 % concordance. Inconsistent data were excluded from the final analysis.

### Sequencing

Ten of APOE rs7412 CC type, CT type and rs429358 TT type, TC type were randomly selected for sequencing, respectively. Primers were designed using Primer premier 5.0 software and were synthesized by Beijing Sunbiotech Biological Engineering Co., Ltd. The primers sequence was shown in Table 5. PCR amplification was performed in C1000TM Thermal Cycler PCR instrument. 30 μl of PCR reaction system contained 15 μl of PCR Master mix (2×), 2.4ul of upstream and downstream primers (10pmol/ul), 11.4 μl of ddH2O, and 1.2 μl of genomic DNA. Cycling parameters were as follows: 95 °C denaturation for 3 min, 94 °C deformation for 30 s, 62 °C annealing for 35 s, 72 °C extension for 40s, a total of 35 cycles were include, total extension at 72 °C for 5 min. After amplification, the PCR products were detected by 1.5 % agarose gel electrophoresis. Direct sequencing by Beijing Sunbiotech Biological Engineering Co., Ltd. was performed to confirm these genotypes.

**Table 5:** The loci, primer sequences, annealing temperature and the product length of amplified gene

Gene locus	Primer Sequences (5’-3’)	Annealing temperature (°C)	Product length (bp)
rs7412, rs429358	GGCACGGCTGTCCAAGGA GCCCCGGCCTGGTACAC	62	228

### Statistical analysis

Continuous variables with a normal distribution were reported as means ± standard deviation (SD) and tested by an independent samples *t*-test; continuous variables with a skewed distribution were reported as medians (Q1 and Q3) and tested by the Kolmogorov–Smirnov test; categorical variables were reported as counts (frequencies) and tested by a two-sided chi-square test. The HWE test was conducted to assess whether genotype frequencies were in conformity with predictions based on allele frequencies. PHASE v2.1 software was used to construct haplotypes of *APOE* SNPs rs429358 and rs7412. Warfarin dosage with a skewed distribution was square root-transformed before analysis. Because the E2/E4 genotype is rare, while E2 and E4 alleles have a reciprocal influence on warfarin dose requirements, the four patients with an E2/E4 genotype were excluded from subsequent analysis. Differences in the daily maintenance dose of warfarin and INR values in different genotype groups were calculated using analysis of variance with post hoc comparison using least significant difference analysis. Subsequently patients were divided into low-intensity anticoagulant treatment group (1.6 ≤ INR <2.0) and relatively high-intensity anticoagulant treatment group (2.0 ≤ INR ≤2.5) based on measured INR values. Differences in the daily maintenance dose between E2/E3 heterozygotes and E3/E3 homozygotes were calculated using two independent-samples *t*-tests in both subgroups. Finally, we considered sex, age, BSA, BMI, eGFR categories, smoking and drinking status, coronary artery disease, hyperlipidemia, hypertension, diabetes, stroke history, measured INR, number of concomitant medications that increase INR value, together with *APOE* genotypes as independent variables in the multivariable regression analysis. For *APOE* genotypes, we set the E3/E3 genotype as a reference and regarded E2/E3 and E3/E4 as dummy variables. A two-tailed probability value of <0.05 was considered significant. Statistical analyses were carried out using Statistical Package for Social Science (SPSS ver.20.0, SPSS Science, Chicago, IL).

## Abbreviations

APOE: apolipoprotein E
SNPs: single nucleotide polymorphisms
INR: International normalized ratio
BSA: body surface area
BMI: body mass index
CAD: coronary artery disease
SBP: systolic blood pressure
DBP: diastolic blood pressure
HDL-C: high-density lipoprotein cholesterol
LDL-C: low-density lipoprotein cholesterol
FBG: fasting blood glucose
CRP: C-reactive protein
BUN: blood urea nitrogen
SCR: serum creatinine
eGFR: glomerular filtration rate
HWE: Hardy–Weinberg equilibrium
